# Short-term renal outcomes following acute kidney injury among dengue patients: A follow-up analysis from large prospective cohort

**DOI:** 10.1371/journal.pone.0192510

**Published:** 2018-02-26

**Authors:** Tauqeer Hussain Mallhi, Amer Hayat Khan, Azreen Syazril Adnan, Azmi Sarriff, Yusra Habib Khan, Siew Hua Gan

**Affiliations:** 1 Department of Pharmacy Practice, Faculty of Pharmaceutical Sciences, Government College University Faisalabad, Faisalabad, Pakistan; 2 Discipline of Clinical Pharmacy, School of Pharmaceutical Sciences, Universiti Sains Malaysia, Penang, Malaysia; 3 Chronic Kidney Disease Resource Centre, School of Medical Sciences, Health Campus, Universiti Sains Malaysia, Kubang Kerain, Kelantan, Malaysia; 4 Department of Pharmacy Practice, Institute of Pharmaceutical Sciences, Lahore College for Women University, Lahore, Pakistan; 5 School of Pharmacy, Monash University Malaysia, Bandar Sunway, Malaysia; University of Sao Paulo Medical School, BRAZIL

## Abstract

**Background:**

Despite myriad improvements in the care of dengue patients, acute kidney injury (AKI) remained least appreciated intricacy of dengue infection. Exiting literature does not provide any information on renal outcomes among dengue patients surviving an episode of AKI.

**Methods:**

Dengue patients who developed AKI were followed up for post-discharge period of three months and renal recovery was assessed by using recovery criteria based on different thresholds of serum creatinine (SCr) and estimated glomerular filtration rates (eGFR).

**Results:**

Out of the 526 dengue participants, AKI was developed in 72 (13.7%) patients. Renal recovery was assessed among AKI survivors (n = 71). The use of less (±50% recovery to baseline) to more (±5% recovery to baseline) stringent definitions of renal recovery yielded recovery rates from 88.9% to 2.8% by SCr and 94.4% to 5.6% by eGFR, as renal function biomarkers. At the end of study, eight patients had AKI with AKIN-II (n = 7) and AKIN-III (n = 1). Approximately 50% patients (n = 36/71) with AKI had eGFR primitive to CKD stage 2, while 18.3% (n = 13/71) and 4.2% (n = 3/71) patients had eGFR corresponding to advanced stages of CKD (stage 3 & 4). Factors such as renal insufficiencies at hospital discharge, multiple organ involvements, advance age, female gender and diabetes mellitus were associated with poor renal outcomes.

**Conclusions:**

We conclude that dengue patients with AKI portend unsatisfactory short-term renal outcomes and deserve a careful and longer follow-up, especially under nephrology care.

## Introduction

In recent years with the geographical spread of dengue illness and with more involvement of adults, there have been increasing reports of dengue viral infection (DVI) with unusual manifestations, termed as “Expanded Dengue Syndrome” (EDS). These isolated organopathies include hepatic, renal, cardiac, respiratory and neurological involvements in dengue infection and could be explained as complications of severe profound shock or associated with underlying host conditions or co-infections [1]. Renal involvements in DVI vary from elevation of the serum creatinine (SCr), acute tubular necrosis (ATN), hemolytic uremic syndrome, proteinuria, glomerulopathy, nephrotic syndrome and acute kidney injury (AKI) [2].

Despite notable evidence that transient increase in SCr is linked to increased mortality [3], AKI is still least appreciated and poorly studied complication of DVI. The available data indicate the presence of AKI in 0.83% to 14.2% of dengue patients, depending upon methodology and population being assessed [4–12]. The findings originated from retrospective case series demonstrate that dengue-induced AKI is a highly morbid as well as fatal complication and is associated with prolonged hospitalization [4, 12]. The outcome of AKI if managed properly is fair, but a significant proportion could succumb to it. In a recent case series, we reported approximately half of the dengue patients with AKI diagnosed by the Acute Kidney Injury Network (AKIN) and RIFLE (risk, injury, failure, loss of function, end stage renal disease) criteria and more than 90% of patients diagnosed by the conventional criterion (SCr >2mg/dL) had renal insufficiencies at hospital discharge [13].

The recovery of kidney function following AKI is an important determinant of morbidity and may have long-term implications for the health and well-being of patients [14]; however there has been a dearth of investigation on renal recovery among dengue patients with AKI. The existing literatures merely address epidemiology and predisposing factors of dengue-induced AKI. Furthermore, the evidence of incomplete or partial renal recovery following AKI has already been documented in other infectious diseases including leptospirosis and malaria [15, 16]. To the best of our knowledge, there are no follow up data on renal outcomes in dengue patients. In this context, we carried out prospective single center observational study 1) to assess post-discharge renal recovery by using several criteria and 2) to evaluate factors associated with poor renal outcomes in dengue patients with AKI.

## Methodology

### Ethical approval

The study was approved by Hospital Human Resource Ethics Committee (JEPeM) (USM/JEPeM/14080278) which complies with the Declaration of Helsinki. Patient`s Identity was kept confidential and written informed consents were obtained from all participants. In case where the patients were not able to communicate or in children below 18 years, informed consents were obtained from parents or guardians.

### Study location and population

**Fig 1:**
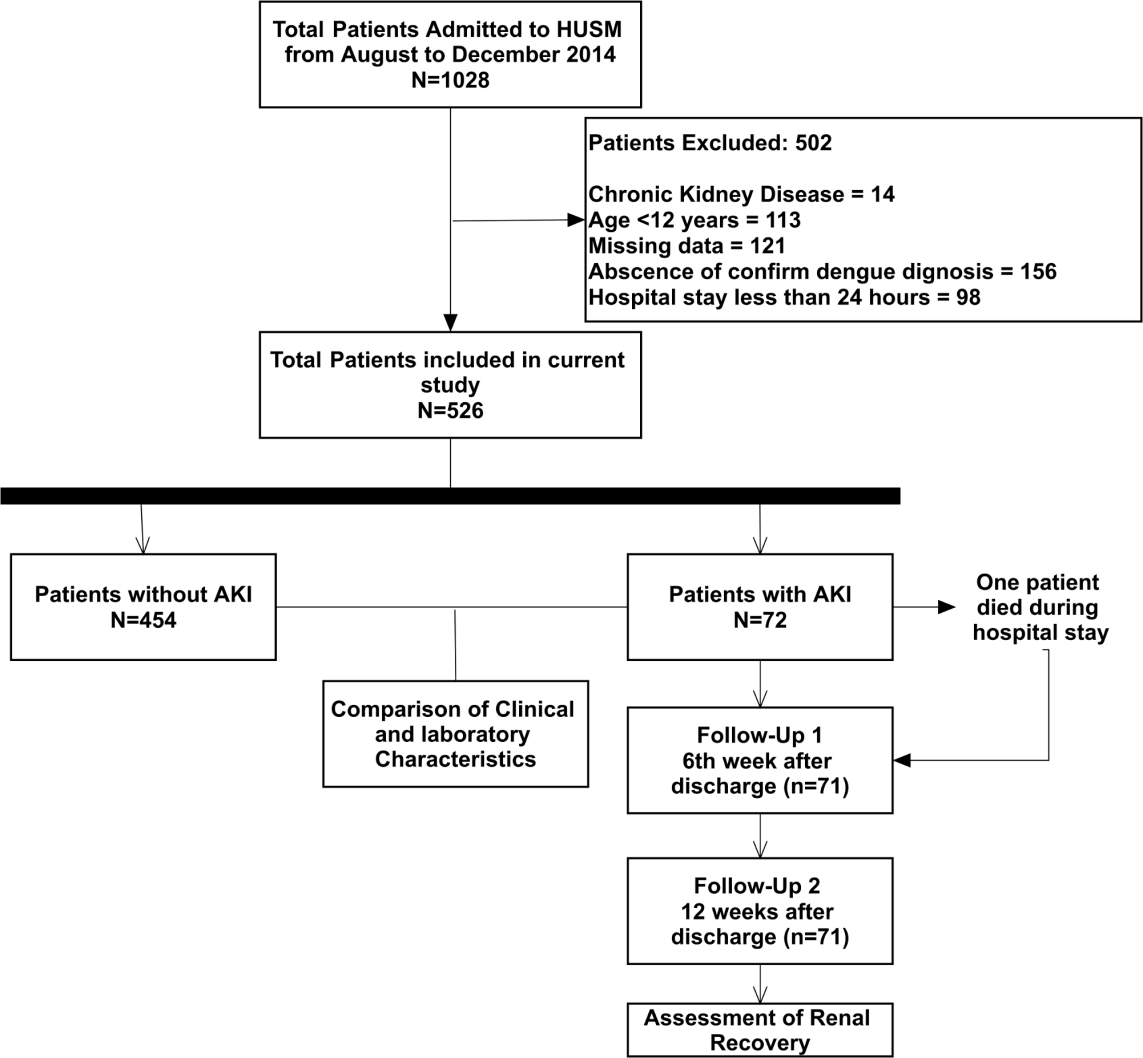
Study flow process.

Current study was conducted in Hospital University Sains Malaysia (HUSM), tertiary level teaching hospital with 950 beds that serves an estimated 1.4 to 1.8 million inhabitants of Kelantan, Malaysia [17]. All the dengue patients admitted to the hospital during August 2014 to December 2014 were included into the study. Patients having age ≥ 12 years admitted with primary and confirmed diagnosis of DVI, irrespective of severity, were identified by using hospital record management system. Dengue patients with AKI were followed up at HUSM Chronic Kidney Disease (CKD) resource center at 6 weeks interval to ascertain renal status for a period of 12 weeks (90 days). Patients with CKD were excluded from the study on account of progressive decline in renal function with the passage of time. On the other hand, patients staying <48 hours in hospital were also excluded to meet the classification of AKI that requires transient increase in SCr within 48 hours. The process of patient’s selection and identification along with inclusion and exclusion criteria are described in Fig 1.

### Diagnosis of dengue

Suspected dengue cases were confirmed by laboratory criteria that were further subjected to clinical case definition of DVI. Suspected cases were confirmed by using at least one of the following criteria: (1) positive reverse transcriptase polymerase chain reaction (RT-PCR) result, (2) presence of dengue immunoglobulin M and G antibodies in acute phase serum by enzyme linked immunosorbent assay [Pan Bio Dengue IgM ELISA, Dengue IgM Dot Enzyme Immunoassay, SD Dengue IgM and IgG capture ELISA Kits; Standard Diagnostics, Korea], and (3) at least 4-fold increase of dengue-specific hemagglutination inhibition titers in convalescent serum when compared with acute phase serum. The serum samples were also tested for dengue-specific NS1 [pan-E Early dengue ELISA kit by Panbio, Australia and Platelia dengue NS1Ag assay by Bio-Rad Laboratories, USA)]. Only confirmed dengue cases were included in the analysis. Primary dengue infection was distinguished from secondary infection by using IgM-IgG ratio where dengue infection was defined as primary if ratio ≥ 1.8 and as secondary if < 1.8 or if there was a 4-fold increase of hemagglutination inhibition and the titers were ≤1:1280 and ≥1:2560, respectively [17]. Serologically confirmed dengue patients were subjected to clinical case definition while disease severity was classified into dengue fever (DF), dengue hemorrhagic fever (DHF) and dengue shock syndrome (DSS) according to the WHO criteria [1].

### Classification of AKI

AKI was stratified by using AKIN criterion and referred to abrupt reduction (within 48 hours) in kidney function as defined by an absolute increase in SCr of ≥26.4 μmol/L or increase in SCr ≥1.5 times from baseline or urine output (UO) < 0.5 ml/kg/h for 6–12 hours. AKI patients were further classified into severity stages i.e. AKIN-1, AKIN-II and AKIN-III (18). We relied on SCr levels for the diagnosis of AKI because of missing data and poor documentation of daily UO among the investigated population. Patients without baseline SCr and having no history of chronic renal insufficiency, baseline SCr was estimated with Modification of Diet in Renal Disease (MDRD) equation by assuming glomerular filtration rate as 75 ml/min/1.73m^2^. Similar approach of the estimating baseline SCr has been used in many studies and has recently been validated [18].

### Data collection and management

All the required data were collected on structured case report form. After identification of confirmed dengue cases, numeral codes were given to patients and these codes were used as identifier during data analysis. Patient`s demographics, clinical presentations and laboratory data were recorded for each patient. The renal status of patients was assessed during each follow-up visit.

### Definitions

Dengue viral infection (DF, DHF, DSS); severe dengue (DHF, DSS); severe AKI (AKIN-III), multiple organ involvements (dysfunction of ≥2 organs other than kidney); hepatic dysfunction (elevation of liver enzymes from ULN); and thrombocytopenia (platelets count < 100×10^9^ cells). Reference values of laboratory parameters in current study are according to the hospital pathology lab included AST (5–34 IU/L); ALT (10–35 IU/L); ALP (♂: 53–168 ♀: 42–98 IU/L); Platelets (158–410×10^9^/L); WBCs (♂:3.8–9.7 ♀: 3.4–10.1); PT (12–13 seconds); aPTT (30–50 seconds).

#### Definition of renal recovery

Definition of renal recovery depends upon the time frame of interest and the study population [19]. We used several criteria to define renal recovery including discontinuation of dialysis, disappearance of AKI criteria, return to ±5, ±10, ±20, ±25 or ±50% of baseline SCr/eGFR and return of SCr/eGFR to pre-AKI values. Estimated glomerular filtration rate (eGFR) was calculated by using CKD-EPI equation (>18 years) and Bedside Schwartz Equation (<18 years). Both SCr and eGFR were used as renal function biomarkers to assess the renal recovery.

### Statistical analysis

For quantitative variables, measures of central tendency and dispersion were calculated. Qualitative variables are presented as frequencies and proportions for which frequency was served as numerator and total number of patients was served as denominator. Relevant denominator was stated before proportion, where it varied. Comparison of categorical variables between two groups was done by using Chi-Square test (if at least 80% of cells have expected frequencies of ≥5) or Fisher`s Exact test (if less than 80% of cells have expected frequencies of ≥5). Comparison of continuous variables was done by an independent Student’s t-test. The two-sided statistical significance level, p-value, was set at 0.05 for all inferential analyses in this study. Data were compiled and analyzed using Statistical Package for Social Sciences program version 20 (SPSS: Inc. Chicago. Il. USA).

## Results

### General characteristics of study population

Out of the total 1028 patients admitted during the study period, 526 patients were enrolled in the current study. Patients who were excluded (N = 502) had CKD (n = 14), age <12 years (n = 113), missing data required for analysis (n = 121), no confirmatory dengue diagnosis (n = 156) and hospital stay of less than 24 hours (n = 98) (Fig 1). The mean age of the study participants was 33.5 ± 16.6 years with male to female ratio of 1.00:1.02. Most patients were urban residents which might be attributed to the locality of the hospital in urban setting. According to WHO dengue classification, 87.1% patients had DF while DHF (Grade I & II) and DSS (Grade III & IV) were observed in 59 (11.2%) and 9 (1.7%) patients, respectively. Ethnic Malays were predominant with 97.3% of total cases followed by Chinese (1.5), Thais (0.8%) and Indians (0.4%).

According to AKIN criterion, 72/526 (13.7%) patients had AKI: 62/72 (86.1%) at AKIN-I, 7/72 (9.7%) at AKIN-II and 3/72 (4.2%) at AKIN-III. A total of 67 patients had AKI on the very first day of hospital admission with AKIN-I (n = 58), AKIN-II (n = 6) and AKIN-III (n = 3). Five patients without any evidence of kidney injury on hospital admission, later developed AKI during hospitalization with AKIN-I in four patients and AKIN-II in one patient. None of the patients with mild degree of AKI progressed to severe AKI during hospitalization.

Overall, the mortality rate in current study was 0.4% (n = 2). The documented causes of death were septic shock, aspiration pneumonia, meningoencephalitis, dehydration secondary to dengue fever in one patient, while severe AKI, shock, altered mental status and coagulopathy in other patient. One fatal case had AKI and hence not included in the follow-up analysis. The pattern of renal recovery during and after hospital discharge was evaluated by using several criteria based on SCr and eGFR (Table 1). Fortunately, none of the patients required dialysis during hospitalization, therefore criterion “discontinuation of dialysis” was not used in current study. A total 71 AKI survivors were followed up at intervals of 6 weeks (Follow-Up 1) and 12 weeks (Follow-Up 2).

**Table 1:** Pattern of renal recovery among 71 dengue patients with AKI at discharge, 6 weeks (Follow-up 1) and 12 weeks (Follow-up 2) post-discharge interval by using different criteria of renal recovery.

	Full renal Recovery n (%)		± 5% recovery to baseline n (%)		± 10% recovery to baseline n (%)		± 20% recovery to baseline n (%)		± 25% recovery to baseline n (%)		± 50% recovery to baseline n (%)		Disappearance of AKI n (%)	
	Yes	No	Yes	No	Yes	No	Yes	No	Yes	No	Yes	No	Yes	No
**On the Basis of Serum Creatinine (SCr)**														
**On discharge**	19 (26.8%)	52 (73.2%)	28 (39.4%)	43 (60.6%)	41 (57.7%)	30 (42.3%)	50 (70.4%)	21 (29.6%)	57 (80.3%)	14 (19.7%)	67 (94.4%)	4 (5.6%)	67 (94.4%)	4^a^ (5.6%)
**Follow-up 1**	0	71 (100%)	7 (9.9%)	64 (90.1%)	22 (31%)	49 (69%)	43 (60.6%)	28 (39.4%)	49 (69%)	22 (31%)	63 (88.7%)	8 (11.3%)	61 (85.9%)	10^b^ (14.1%)
**Follow-up 2**	0	71 (100%)	2 (2.8%)	69 (97.2%)	5 (7%)	66 (93%)	28 (39.4%)	43 (60.6%)	34 (47.9%)	37 (52.1%)	63 (88.7%)	7 (9.9%)	63 (88.7%)	8^c^ (11.3%)
**On the basis of eGFR**														
**On discharge**	21 (29.6%)	50 (100%)	31 (43.7%)	40 (56.3%)	45 (63.4%)	26 (36.6%)	56 (78.9%)	15 (21.1%)	63 (88.7%)	8 (11.3%)	69 (97.2%)	2 (2.8%)	-	-
**Follow-up 1**	0	71 (100%)	12 (16.9%)	59 (83.1%)	25 (35.2%)	46 (64.8%)	47 (66.2%)	24 (33.8%)	52 (73.2%)	19 (26.8%)	70 (98.6%)	1 (1.4%)	-	-
**Follow-up 2**	0	71 (100%)	4 (5.6%)	67 (94%)	12 (16.9%)	59 (83.1%)	31 (43.7%)	40 (56.3%)	45 (63.4%)	26 (36.6%)	67 (94.4%)	4 (5.6%)	-	-

^a^Four patients who had AKI on discharge were having AKIN-II

^b^Ten patients with AKI on first follow-up: AKIN-I in 2 patients, AKIN-II in 8 patients

^c^Eight patients with AKI on second follow-up: AKIN-II in 7 patients, AKIN-III in 1 patient

### Renal recovery during hospital discharge

**Fig 2:**
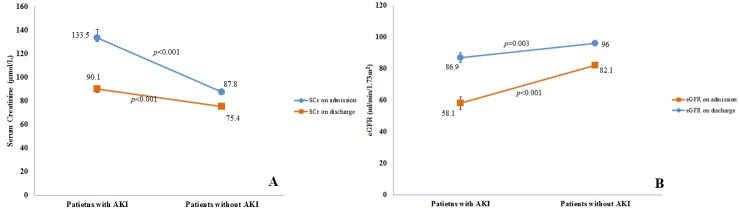
Mean values of SCr (A) and eGFR (B) among dengue patients with and without AKI at hospital admission and discharge.

During hospital discharge, patients with AKI had significantly higher SCr (75.4 ± 16.3 vs 90.9 ± 24.7, *p*<0.001) and lower eGFR levels (95.9 ± 19.8 vs 86.1 ± 25.6, *p*<0.001) than patients without AKI (Fig 2). Table 1 indicates that only 26.8% (n = 19/71) patients recovered their pre-AKI SCr at hospital discharge. Twenty one (29.6%) and 14 (19.7) patients had non-recovery even with less stringent definitions i.e. 20% and 25% return to baseline SCr, respectively. Four patients had AKI (AKIN-II) at the time of discharge (Table 1).

### Pattern of renal recovery during follow-up

**Fig 3:**
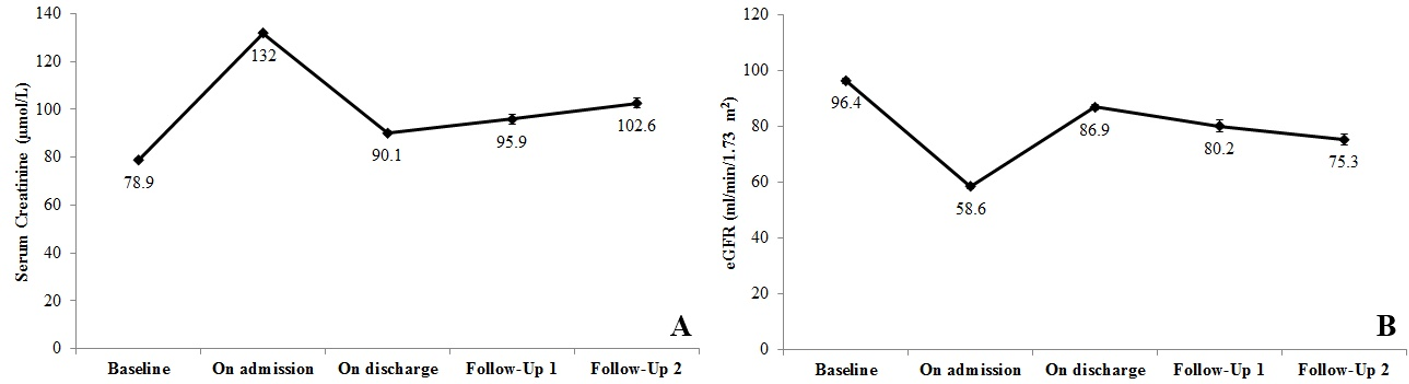
Mean values of serum creatinine (A) and eGFR (B) with standard errors in patients with AKI during follow-up periods (On admission, after 6 weeks (follow-up 1) and after 12 weeks (follow-up 2).

The use of the different recovery criteria led to a wide variation in the rate of renal recovery, where relaxing the criterion caused gradual increase in proportion of patients who recovered their kidney function (Table 1). When most stringent threshold corresponding to the return of SCr/eGFR to baseline (pre-AKI values) was used, none of the patient demonstrated renal recovery upon completion of follow-up. Fig 3 presents fluctuations of SCr and eGFR during study process. A gradual increase in SCr and decrease in eGFR was observed during follow-up period (Fig 3).

### Renal recovery by using serum creatinine based definitions

By using SCr as renal biomarker, the rate of renal recovery at the end of follow-up varied between 2.8% and 88.7%, depending upon the recovery criterion used (±5% to ±50% return to baseline) (Table 1). “Disappearance of AKI” criterion was observed in the majority (88.7%) of the patients, while eight patients had AKI at the end of follow-up. Of these, 7 patients had AKIN-II and one patient had AKIN-III stage. Two patients with AKIN-II during hospitalization maintained similar severity of AKI upon completion of their follow-up. Moreover, one patient with AKIN-III during hospitalization improved to AKIN-II while five patients with AKIN-I progressed to AKIN-II (n = 4) and AKIN-III (n = 1), resulting in total eight patients with AKI at the end of study. Other causes of increased SCr were also ruled out during follow-ups with no other factors seen to be associated with SCr elevation.

### Renal recovery by using eGFR based definitions

**Fig 4:**
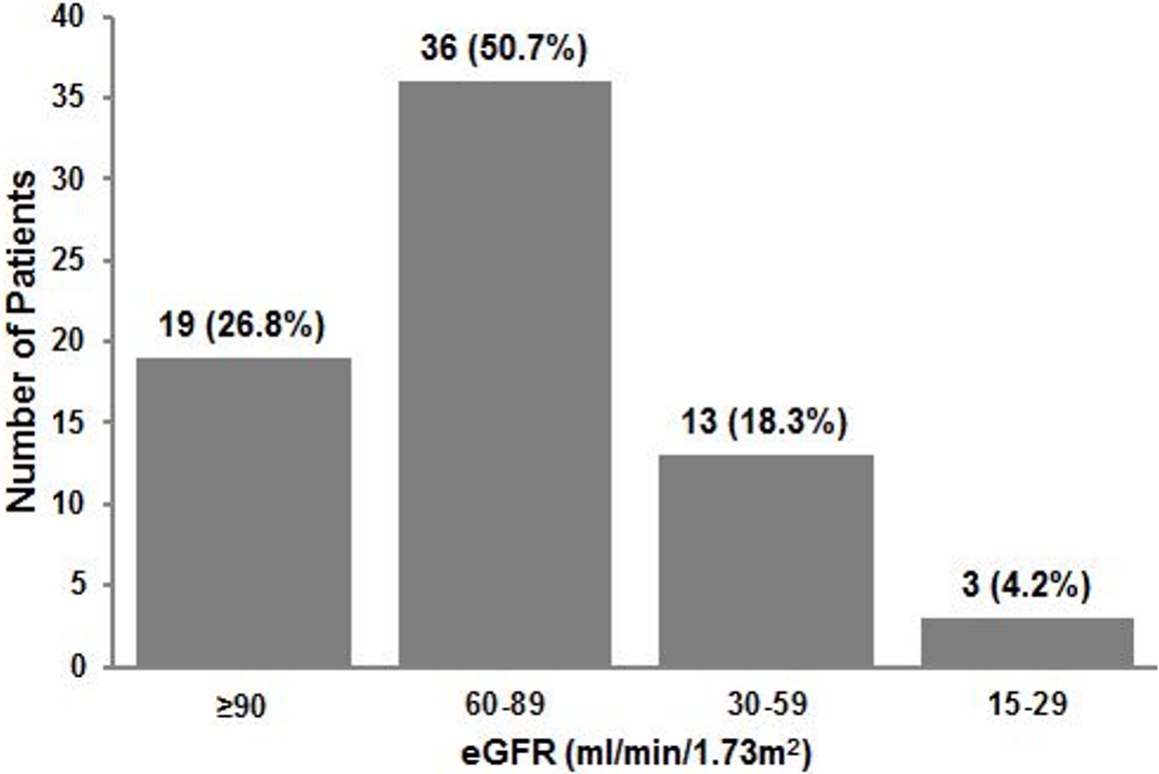
Categorization of patients according to eGFR category at the end of study.

When recovery among AKI survivors was assessed by using eGFR based criteria, the rate of renal recovery was comparatively higher (5.6% to 94.4%) than estimated by SCr based definitions. The mean eGFR at the end of follow-up was 73.3 ± 24.3 ml/min/1.73m^2^. Fig 4 illustrates that 22.5% (n = 16/71) patients had eGFR <60 ml/min/1.73m^2^ while the proportion of patients with eGFR ≥90 ml/min/1.73m^2^ was 26.7%. Subgroup analysis on eight patients who had AKI at the end of study was performed. The mean eGFR among these patients was 44.3 ± 20.3 ml/min/1.73m^2^ with six patients having eGFR <60 ml/min/1.73m^2^.

### Urinalysis of AKI survivors during follow-up

All patients who were able to provide urine sample underwent urinalysis. Out of the 71 patients who had AKI, dipstick positive glycosuria and proteinuria was present in two (2.8%) and 14 (19.7) patients, respectively. Microscopic hematuria and pyuria were also present among substantial number of patients at the end of follow-up period (Table 2). Subgroup analysis on urine examination of eight patients who had AKI at the end of study demonstrated the presence of trace glucose and +1 protein (n = 6), WBCs 0–3 cells/HPF (n = 7), RBCs 0–5 cells/HPF (n = 4) and epithelial cells (n = 7).

**Table 2:** Urinalysis (Dipstick and microscopic) among patients with AKI during follow-up period.

Urinalysis	Results	Follow-up 1^a^	Follow-up 2^b^
Urine Glucose	Negative	64 (90.1%)	58 (81.7%)
	Trace	5 (7%)	11 (15.5%)
	1+	1 (1.4%)	1 (1.4%)
	3+	1 (1.4%)	1 (1.4%)
Urine Protein	Negative	41 (57.7%)	41 (57.7%)
	Trace	17 (23.9%)	16 (22.5%)
	1+	13 (18.3%)	14 (19.7%)
Urine WBCs	Negative	4 (5.6%)	4 (5.6%)
	0–1 cells/HPF	41 (57.7%)	41 (57.7%)
	1–3 cells/HPF	25 (35.2%)	25 (35.2%)
	1–5 cells/HPF	1 (1.4%)	1 (1.4%)
Urine RBCs	Negative	15 (21.1%)	15 (21.1%)
	0–1 cells/HPF	33 (46.5%)	33 (46.5%)
	1–3 cells/HPF	15 (21.1%)	15 (21.1%)
	1–5 cells/HPF	8 (11.3%)	8 (11.3%)
Urine Epithelial cells	Negative	24 (33.8%)	22 (31%)
	1–3 cells/HPF	46 (64.8%)	48 (67.6%)
	1–5 cells/HPF	1 (1.4%)	1 (1.4%)

^a^6^th^ week after discharge

^b^12^th^ week after discharge

Abbreviations: HPF (high power field)

### Clinico-laboratory factors associated with non-recovery after AKI

To determine the factors associated with non-recovery after dengue-induced AKI, we compared patients with and without renal recovery by using three SCr based recovery criteria (Table 3). The criteria “±5% and ±10% return to baseline SCr” were not used for the statistical comparison due to very few number of patients who did not recover (n = 2 and n = 5 respectively). The criterion “50% return to baseline SCr” corresponds to “disappearance of AKI” criterion and was therefore not included in the analysis. Table 3 demonstrates that AKIN-I stage of AKI was associated with renal recovery while the presence of MOIs during hospitalization and non-recovery at hospital discharge were significantly associated with non-recovery based on ±20% and ±25% criteria of renal recovery. When diagnosis of renal recovery was made with “disappearance of AKI” criterion, the factors associated with non-recovery were advance age, female gender, diabetes mellitus, use of nephrotoxic drugs, secondary infection, non-recovery or eGFR <60 ml/min/1.73m^2^ during hospital discharge. Irrespective to the recovery criteria, most of the patients who did not recover were >60 years old, had DSS, severe stages of AKI, rhabdomyolosis and warning signs during their hospitalizations. However, these factors lack statistically significant association with non-recovery in our analysis (Table 3).

**Table 3:** Clinico-laboratory characteristics (during hospitalization) of AKI patients without renal recovery at the end of follow-up period (12 weeks) by using SCr as renal function biomarker.

	Total patients	± 20% return of SCr to baseline			± 25% return of SCr to baseline			Disappearance of AKI		
	N = 71	Recovered N = 28	Not recovered N = 43	P values	Recovered N = 34	Not recovered N = 37	P values	Recovered N = 63	Not recovered N = 8	P values
**Age (years, mean ± SD)**	39.6 ± 17.8	36.8 ± 18.1	41.4 ± 17.6	0.284	37.3 ± 18.1	41.7 ± 17.5	0.303	37.6 ± 17.1	55.6 ± 15.7	**0.006**
Age> 60 years, n (%)	10 (14.1%)	3 (10.7%)	7 (16.3%)	0.730	4 (11.8%)	6 (16.2%)	0.590	8 (12.7%)	2 (25.0%)	0.346
Female	24 (33.8%)	11 (60.7%)	13 (69.8%)	0.431	11 (32.4%)	13 (35.1)	0.804	17 (27%)	7 (87.5%)	**0.001**
DHF (Grade I & II), n (%)	9 (12.7%)	6 (21.4%)	3 (7.0%)	0.074	7 (20.6%)	2 (5.4%)	0.077	7 (11.1%)	2 (25.0%)	0.266
DHF (Grade II & III), n (%)	5 (7%)	1 (3.6%)	4 (9.3%)	0.642	1 (2.9%)	4 (10.8%)	0.359	4 (6.3%)	1(12.5%)	0.460
**AKI Classification**										
AKIN-I, n (%)	62 (87.3%)	28 (100%)	34 (79.1%)	**0.009**	33 (97.1%)	28 (78.4%)	**0.029**	57 (90.5%)	5 (62.5%)	0.058
AKIN-II, n (%)	6 (8.5%)	0	6 (14.0%)	0.075	1 (2.9%)	5 (13.5%)	0.201	4 (6.4%)	2 (25.0%)	0.133
AKIN-III, n (%)	3 (4.2%)	0	3 (7.0%)	0.273	0	3 (8.1%)	0.241	2 (3.2%)	1 (12.5%)	0.305
**Comorbid conditions**										
Hypertension, n (%)	13 (18.3%)	5 (17.9%)	8 (18.6%)	0.938	7 (20.6%)	6 (16.2%)	0.634	10 (15.9%)	3 (37.5%)	0.156
Diabetes mellitus, n (%)	10 (14.1%)	2 (7.1%)	8 (18.6%)	0.296	2 (5.9%)	8 (21.6%)	0.87	6 (9.5%)	4 (50.0%)	**0.011**
Hyperlipidemia, n (%)	4 (5.6%)	2 (7.1%)	2 (4.7%)	0.644	2 (5.9%)	2 (5.4%)	1.000	3 (4.8%)	1 (12.5%)	0.387
Ischemic heart disease, n (%)	5 (7%)	1 (3.2%)	4 (9.3%)	0.642	1 (2.9%)	4 (10.8%)	0.359	4 (6.4%)	1 (12.5%)	0.460
Multiple organ involvements, n (%)	14 (19.7%)	1 (3.6%)	13 (30.2%)	**0.006**	3 (8.8%)	11 (29.7%)	**0.027**	11 (17.4%)	3 (37.5%)	0.186
Rhabdomyolosis, n (%)	4 (5.6%)	0	4 (9.3%)	0.148	0	4 (10.8%)	0.116	2 (3.2%)	2 (25.0%)	0.060
Use of nephrotoxic drugs, n (%)	18 (25.4%)	6 (21.6%)	12 (27.9%)	0.540	7 (20.6%)	11 (29.7%)	0.376	12 (19%)	6 (75.0%)	**0.003**
Secondary Infection	10 (14.1%)	12 (42.2%)	21 (48.8)	0.296	3 (8.8%)	7 (18.9%)	0.311	6 (9.5%)	4 (50.0%)	**0.011**
Warning signs	33 (46.4%)	2 (7.1)	8 (18.6)	0.621	13 (38.2%)	20 (54.1%)	0.182	28 (44.4%)	5 (62.5%)	0.459
Delayed hospitalization^€^	21 (29.6%)	8 (28.8%)	13 (30.2%)	0.229	11 (32.4%)	10 (27%)	0.362	18 (28.8%)	3 (37.5)	0.258
No recovery on discharge*, n (%)	-	1 (3.6%)	20 (46.5%)	**<0.001**	1 (2.9)	13 (35.1%)	**<0.001**	7 (11.1%)	5 (62.5%)	**0.004**
eGFR<60 ml/min/1.73m^2^ (at discharge)	12 (16.9%)	2 (7.1%)	10 (23.3%)	0.108	4 (11.8%)	8 (21.6%)	0.268	7 (11.7%)	5 (62.5%)	**0.003**
SCr, μmol/L (at discharge)	90.1 ± 23.9	80.8 ± 17.7	96.3 ± 25.6	**0.007**	84.6 ± 20.9	95.2 ± 25.6	0.060	87.3 ± 18.7	112.5 ± 44.2	0.153
eGFR, ml/min/1.73m^2^ (at discharge)	86.9 ± 25.3	95.8 ± 21.6	81.0 ± 26.0	**0.015**	93.8 ± 24.1	80.5 ± 25.0	**0.025**	89.9 ± 22.5	63.1 ± 34.1	0.064
SCr, μmol/L (at the end of follow-up)	102.6 ± 27.2	87.2 ± 18.2	112.7 ± 27.5	**<0.001**	91.6 ± 21.6	112.8 ± 28.1	**0.001**	98.1 ± 20.2	138.3 ± 46.5	**0.045**
eGFR (at the end of follow-up)	73.3 ± 24.3	88.2 ± 22.5	66.7 ± 21.8	**<0.001**	86.4 ± 23.8	65.1 ± 20.2	**<0.001**	79.2 ± 21.9	44.3 ± 20.3	**<0.001**

Abbreviations: DHF: dengue hemorrhagic fever, AKI: acute kidney injury, eGFR: estimated glomerular filtration rate, SCr: serum creatinine

*renal recovery at discharge by using corresponding criterion

^€^Hospital admission ≥5 days of onset of illness

## Discussion

To our knowledge, this is a first study to demonstrate the pattern of renal recovery in representative heterogeneous cohort of dengue patients attending a tertiary level teaching hospital. AKI was a least appreciated complication of DVI prior to the development and validation of the first standardized multi-dimensional AKI definition known as the RIFLE criterion, and its subsequent recalibration, AKIN and KDIGO criteria [19]. Our analysis stratified 72 (13.7%) patients with AKI on the basis of AKIN classification, where AKIN-I constitutes most of the cases (n = 62/72). Previously, dengue-induced AKI was defined by several criteria [4–13] that led to great disparity in its incidence as well as epidemiology, making it difficult or even impossible to compare findings across these studies. From our observation, the incidence of AKI in the present study is comparable to those that stratified AKI with AKIN criterion (4, 5) but is slightly higher (0.9% to 3.3%) than other studies using stringent AKI definition as rise of SCr ≥176.8 μmol/L or ≥2 mg/dl (7, 8). It is well documented that transient increase in serum creatinine is linked to the increased mortality [3, 20], therefore the use of stringent criteria may exclude mild AKI cases (AKIN-I) which accounted for the majority (86.1%) of AKI cohort in the present study. However, even with the use of similar criteria the incidence of AKI in dengue infection may vary. This varying incidence might be attributable to several factors including study design, biasness in patient`s selection, heterogeneity of studied population, severity of dengue, lack of consensus to define AKI, availability of baseline SCr and time of evaluation. Since a significant number of dengue patients experience AKI, it is suggested that there is a dire need to develop a consensus to produce a proper definition of AKI in dengue infection.

Despite a growing body of evidence of increased morbidity and mortality associated with AKI caused by dengue infection, the data on renal recovery and post-AKI epidemiology is currently lacking. To the best of our knowledge, only one study followed up nine survivors of dengue-induced AKI and reported that SCr levels returned to normal in 32 days [7]. However, this study was accompanied by several limitations, which are primarily low sample size and inclusion of only children (age<15 years) with severe forms of dengue infection (DHF/DSS). In this regard, current study is the first to demonstrate post-AKI epidemiology during the follow-up period of 90 days.

Most of the studies addressing renal recovery include critically ill patients requiring dialysis or severe forms of AKI and considers renal recovery as dialysis independency [21, 22]. However, milder forms of AKI not requiring dialysis are also associated with high morbidity and mortality, and failure to recover from these stages would greatly impact long-term outcomes. Indeed, for many, this would mean worsening of underlying CKD or *de-novo* CKD, with all the attendant morbidity [23, 24]. However, little is known about renal recovery and its influence on the outcomes in these patients. In the current study, mild AKI (AKIN-I) was observed in 62 patients and none recovered their pre-AKI SCr at the end of follow-up period. Of these, only 34 and 28 patients recovered their 20% and 25% baseline SCr, respectively (Table 3). Alarmingly, five patients with mild AKI during hospitalization progressed to severe stages at the end of study. These findings suggest that failure to recover normal kidney functions also occurs even in milder forms of AKI.

Today, studies on AKI recovery suffer from the same issue as research on AKI had several years ago, when a consensual definition was lacking. The use of different criteria of recovery precludes any synthetic analysis of the literature and demonstrates a wide range of post-AKI non-recovery [25]. Most of the studies reporting post-AKI epidemiology were conducted on critically ill patients where values of SCr are confounded by several factors. Therefore, their methodological implications cannot be applied to the studies evaluating renal recovery among less severe patients. These patients may also require alternate definition for assessing renal recovery [26]. Moreover, the definition for renal recovery should also include a standardized time point [19]. In the present study, renal function was assessed by SCr measured after 90 days of AKI episode to allow sufficient time for recovery. The stipulated time frame is also in accordance with KDOQI guidelines. Since renal recovery should be assessed among survivors, therefore one patient with AKI who passed away was excluded from the analysis (Tables 1–3) [27]. All the patients were discharged by using standard criteria including afebrile for 48 hours, improved clinical status (general well-being, appetite, hemodynamic status, urine output, no respiratory distress), increasing trend of platelet count, stable hematocrit without intravenous support with clinical adjudication of attending physicians.

Several relaxed to more stringent criteria of renal recovery based on SCr and eGFR threshold were used in current study (Table 1). At hospital discharge, 26.8% and 29.6% patients recovered their pre-AKI SCr and eGFR respectively. However, recovery of pre-AKI SCr and eGFR was not observed during first and second follow-up. It is interesting to note that by using 5% to 25% recovery to baseline SCr, the rate of renal recovery varied from 9.9% to 69% at first follow-up that was subsequently reduced to 2.8% to 47.9% during second follow-up. Likewise, criteria 5% to 25% recovery to baseline eGFR resulted in recovery rate ranging from 16.9% to 73.2% (first follow-up) and 5.6% to 63.4% (second follow-up). It is noteworthy that the rate of renal recovery estimated by eGFR based definitions was higher than did by SCr based definitions. It might be attributed to the reason that GFR estimation reflects late functional changes, which are inherently delayed since the kidneys contain the innate ability to maintain function by hyperfiltration and compensatory hypertrophy of the remaining healthy nephrons [19]. The overestimation of rate of renal recovery by using eGFR has recently been reported [28].

The rate of renal recovery gradually increased with less stringent definitions of recovery. These findings are in concordance with the report by Schetz *et al* in which the use of less to more stringent renal recovery criteria causes a shift from complete to partial or non-recovery [27]. In a population based study, Ali *et al*. classified renal recovery of AKI as complete (return of SCr to baseline), partial (SCr above baseline) or non-recovery (presence of AKI) [29]. By applying similar classification to our cohort, none of the patient had complete recovery in the current study while 88.7% (n = 63/71) and 11.3% (n = 8/71) had partial and non-recovery, respectively (Table 3). Although the “disappearance of AKI” criterion is less strict as compared to others but defining recovery from a disease as “absence of disease” seems to be the most logical approach [27]. In the present study cohort, AKI was present both during hospital discharge and at the end of follow-up. Our data indicate a great disparity in the recovery rate by using different criteria. However, whether the difference in the recovery rate by using several definitions has implications for long term outcomes or not, remains to be investigated.

It is worth mentioning that substantial number of patients at the end of study had eGFR values that correspond to the KDIGO classification of CKD (Fig 4). Of these, 50.7% (n = 36/71) patients had residual renal impairment compatible to CKD stage 2, while 22.5% (n = 16/71) patients had eGFR corresponding to the advanced CKD stages such as stage 3 and stage 4 (Fig 4). It is plausible that extended longitudinal follow-up of these patients may result in further improvement in eGFR. Although patients with CKD are excluded from the study but the possibility of uncovering pre-existing unknown CKD by an episode of AKI cannot be ignored. However, the probability of unidentified CKD is remote in the current study due to the presence of an online alert system in our institution. This system identifies hospitalized suspected CKD cases on the basis of eGFR values. For this purpose, a separate unit named “CKD resource center” has been established where identified cases are immediately followed up by renal team to confirm CKD diagnosis. Fortunately there was no report of pre-existing CKD among investigated population. However in any case, *de-novo* CKD diagnosis or the detection of unknown pre-exiting CKD would require similar management with a referral to nephrologists when needed. Though, urine profile along with eGFR at the end of follow-up indicated the presence of suspected *de-novo* CKD but we are unable to draw any firm conclusion due to absence of appropriate CKD diagnosis in the current study including kidney morphology and histology. We strongly believe that the diagnosis of CKD should not merely be based on eGFR since evidence from urine microscopy, kidney morphology and renal biomarkers is of paramount importance [30]. Therefore, we cautiously suggest non-recovery of renal function relating to CKD among dengue patients with AKI. Further studies with appropriate CKD diagnosis are needed to evaluate these findings. However, the most meaningful outcome of this study from a patient`s perspective and for determining the need for nephrologist`s follow-up is the presence of substantial proportion of patients who have no renal recovery.

Non-recovery of kidney function following an episode of AKI is a major morbid event with long term implications on patients and health resources. Unfortunately, none of the studies on dengue-induced AKI has specifically investigated factors predictive of non-renal recovery. Among the clinical variables assessed, adverse renal outcomes during hospital discharge were primarily associated with non-renal recovery regardless of the recovery criteria adopted (Table 3). Compared to the patients who had renal recovery, the SCr and eGFR values were significantly higher in patients who did not recover their kidney function during hospital discharge. Our results indicated that dengue patients who had renal insufficiencies during hospital discharge portended adverse renal outcomes, which are in concordance with the findings of Macedo *et al* [22]. Interestingly, a substantial proportion of patients with AKIN-I also had non-recovery with either criterion (Table 3) and these findings are persistent with reports which indicated adverse renal outcomes in mild AKI not requiring dialysis [23, 24]. By using “disappearance of AKI” criterion, non-recovery was significantly associated with advance age and diabetes mellitus. The association of advanced age and comorbidities with deterioration in renal function has also been well documented by the various studies [31]. Approximately 25% patients with AKI were utilizing nephrotoxic drugs for underlying comorbidities with more profound use among patients without renal recovery (Table 3). Nevertheless, the ongoing exposure of potential nephrotoxic agents predisposes the progression of kidney disease and therefore their use should be discouraged [30]. It is noteworthy that seven out of eight patients, who had AKI upon study completion, were female. Though, gender impact on renal recovery is rarely assessed but few studies reported less recovery among females [14, 31]. In addition, several other differences were also observed between participants who recovered their kidney function versus those who did not, but they lack statistically insignificant association with non-recovery i.e. severe dengue, AKIN-II/III, rhabdomyolysis and warning signs. Considered together, factors contributing to non-recovery in the current study are rather similar to those reported in previous literature on AKI outcomes. We tend to agree with the supposition that identifying patients at-risk during hospital discharge using commonly available characteristics may allow for timely implementation of appropriate screening strategies or surveillance. It is believed that the development of clinical predictive models will be extremely useful in prognostication of adverse renal outcomes following AKI in dengue which may be extended to other infections.

To date, the underlying cellular and physiological mechanisms that drive outcomes following AKI are poorly characterized. Hypothesized mechanisms underlying adverse renal outcomes following AKI include effects of systemic and intra-renal hypertension and hyperfiltration, tubular hypertrophy, and hypertension resulting in arteriosclerosis, tubule-interstitial fibrosis, and glomerulosclerosis, as well as the effects of derangements of the endocrine response and abnormalities in circulating mediators associated with decrements in renal function [32]. Since, infectious diseases cause intra-renal AKI and there are some data to suggest that intra-renal AKI is associated with poor renal recovery as compared to pre or post AKI [33] which may contribute to the fact that most patients with AKI had poor renal recovery as seen in our study. However, this assumption can only be confirmed by conducting renal histology and biopsy in future studies.

Dengue induced AKI is poorly studied intricacy and very little is known about its etiopathogenesis. A recent systemic review described several underlying mechanisms including deposition of immune complex in glomerular mesangium, hemolytic uremic syndrome, multiple organ failure, rhabdomyolysis or myositis and direct viral invasion [34]. Determination of exact mechanism is rather difficult, even in prospective studies. However, we recommend histopathological investigations in order to ascertain the pathophysiological perpetrators of AKI. Since there is no specific treatment for AKI, patients were managed with adequate fluid resuscitation, correction of coagulopathies, avoidance of nephrotoxic agents, inotropes and non-adrenalin support.

Biomarkers of AKI that are capable of early detection, risk stratification, and prognostication would represent a tremendous advance in the care of patients with dengue-induced AKI. Neutrophil Gelatinase associated Lipocalin (NGAL), Kidney Injury Molecule-1 (KIM-1), Liver-type Fatty Acid Binding Protein (L-FABP), Interleukin-18 (IL-18) and NF-κB are some novel AKI biomarkers that provide early and specific diagnosis of AKI with good predictive ability of clinical outcomes [34]. The value of these biomarkers has not been studied in dengue patients. These findings suggest the need of more clinical studies using biomarkers in order to get accurate and robust predictive model of dengue-induced AKI and to ascertain the recovery pattern following injury.

### Study limitations

Being a single-center study, some potential limitations are possible. First, current study relied on SCr criterion for AKI classification, since daily UO data were unavailable for most of the study participants. Therefore, it is plausible that patients who have maximum AKI with oliguria criterion might experience a different pattern of recovery. At the same time, it is unknown if the inclusion of UO criterion may have changed the findings since it has been documented that patients defined by SCr criterion were more severely ill than those defined by the UO criteria [35]. Second, baseline SCr was not available in 143 patients including 11 patients with AKI and therefore estimated by using MDRD equation. Estimating baseline SCr with MDRD has widely been validated and extensively used in existing literature (18). Third, the number of patients without recovery was small making the statistical power weak to perform a logistic regression. Fourth, AKI patients were followed up for three months and there is possibility that further renal recovery occurs with a longer follow-up. However, evaluation of renal recovery at three months following AKI is recommended by KDIGO and has been used in various investigations [18, 30, 36]. Since Macedo *et al*. [22] concluded that renal recovery must be evaluated no earlier than one year; we recommend future studies with longer follow-up for the ascertainment of renal status among dengue patients surviving an episode of AKI. Last but not least, current study lacks information on kidney morphology and histology.

Nevertheless, despite notable limitations, the current study is strengthened by the first report on pattern of renal recovery in dengue infection, utilized a consensus definition for AKI stratification, is a prospective design containing heterogeneous population and has readily available baseline SCr in most of the patients. Additionally, several criteria of renal recovery were utilized so that future studies could be compared with our findings. Since the burden of CKD in tropics is large and the etiology remains unclear in 16% patients [34], it is hoped that the current study can provide the basis for identification of infectious CKD in the future. Present study will provide future implications for the development of consensus on more concrete definition of renal recovery and best the time to assess renal function following infectious AKI.

## Conclusions

Our study demonstrated the high proportion of AKI and its association with a substantial risk of renal deterioration in patients. Depending on the criteria used, the pattern of renal recovery varies among AKI survivors with most patients failing to achieve even 25% of baseline SCr. A significant proportion of patients have eGFR compatible with the criteria of CKD. Overall, presence of factors such as renal insufficiencies during hospital discharge, MOIs, advance age, female gender and diabetes mellitus are associated with short-term adverse renal outcomes. Our data suggest that dengue patients with AKI deserve a careful and long-term medical follow-up, especially under nephrology care. We urge the need for appropriately powered multi-center trials with longer follow-up period to validate our findings and to clarify the utility of criteria for renal recovery in dengue patients.
